# Detection of Postoperative Intestinal Ischemia in Small Bowel Transplants

**DOI:** 10.1155/2012/970630

**Published:** 2012-11-04

**Authors:** Hanne Birke-Sorensen

**Affiliations:** Institute of Clinical Medicine, Aarhus University Hospital, Brendstrupgaardsvej 100, 8200 Aarhus N, Denmark

## Abstract

Small bowel transplantation is acknowledged as auto- and allotransplantation. In both instances, there is up to a 4%–10% risk of postoperative ischemia, and as the small bowel is extremely susceptible to ischemia, the timely diagnosis of ischemia is important. The location of the transplant, whether it is buried in the abdominal cavity or in the neck region, increases the challenge, as monitoring becomes more difficult and the consequences of neglect more dangerous. All methods for the early detection of postoperative ischemia in small bowel transplants are described together with the requirements of the ideal monitoring method. A small bowel transplant can be inspected directly or indirectly; the blood flow can be monitored by Doppler or by photoplethysmography, and the consequences of the blood flow can be monitored. The ideal monitoring method should be reliable, fast, minimally invasive, safe, objective, easy, cheap, and comfortable. No monitoring methods today fulfill the criteria of the ideal monitoring method, and evidence-based guidelines regarding postoperative monitoring cannot be made. The choice of whether to implement monitoring of ischemia—and if so, which method to choose—has to be made by the individual surgeon or center.

## 1. Introduction

Small bowel transplantation (SBT) is an acknowledged therapy as autotransplant for the reconstruction of the upper gastrointestinal tract (mouth, oropharynx, and esophagus) and as an allotransplant for the surgical treatment of short bowel syndrome (pure SBT and in combination with other organs) [1, 2]. 

In both instances, there is up to a 4%–10% risk of postoperative intestinal ischemia in the transplant (POII) [3, 4]. The risk of POII is increased if there is an increased risk of arterial or venous thrombosis, if there is an increased risk of hypoperfusion [5], and if there is an increased risk of compression of the SBT and the vessels after closure of the wound [6]. In auto- as well as allotransplants, the survival of the SBT is of outmost importance, and as the small bowel is extremely susceptible to ischemia, the timely diagnosis of POII is essential. 

Detection of POII in SBT is difficult as the transplant is hidden. The allotransplant is placed intraabdominally and the autotransplant is hidden in the neck region. The abovementioned sensitivity of the SBT in combination with the position of the transplant amplifies the importance of the detection of POII, as neglected POII represents a life-threatening situation. One reason for the importance of detecting POII is that it can aim at an urgent and suitable revascularization to salvage the SBT; another equally important reason is to aim at timely removal of the ischemic SBT to salvage the patient.

Most methods regarding detection of POII are described within autotransplantations, where SBT is one transplant among numerous free tissue transplants (FTTs) used for reconstructive procedures. Since the very start of the era of FTT, and in parallel with the evolution and description of new and more complex FTTs, there has been an ongoing search for methods to allow early detection of any sign of postoperative ischemia. Within allotransplantation, the challenge is not only to make the SBT survive the first 5 days but also to avoid rejection. The focus regarding postoperative monitoring within allo-SBT has therefore been—like in all other allotransplantations—more toward the detection of rejection and malfunction of the SBT. Nevertheless, knowledge obtained in one area can be useful in the other, and the knowledge is still needed, as the rate of vascular thrombosis is reported to be up to 5%–9% for auto- as well as allo-SBTs [3, 4]. 

The goal of this paper is to present all the methods described for the early detection of POII, to describe the advantages and disadvantages of the methods, and to describe the characteristics of the ideal method for the detection of POII in SBT.

## 2. The Monitoring Methods for the Detection of POII

All methods for the detection of POII in SBT can be divided into 3 groups based on the principles of the method.

1. Direct and indirect visualization.
2. Monitoring the blood flow.
3. Monitoring the consequences of the blood flow.

In the following, all the monitoring methods will be described according to these 3 groups.

## 3. Direct and Indirect Visualization 

This group of monitoring methods comprises all methods involving inspection of a part of the SBT, from a simple inspection performed with the naked eye to methods where either special procedures are undertaken to enable simple inspection or special instruments are utilized to allow for or improve the inspection.

### 3.1. Simple Clinical Monitoring

For auto-SBT with an intraoral segment and for allo-SBT with a stoma, simple clinical monitoring of the mucosa can be performed [7, 8]. Color as well as spontaneous and provoked peristaltic movements can be seen by the naked eye. Furthermore, the amounts and color of bleeding after pinprick can be assessed. 

Though this method seems straightforward, it can only be performed by experienced staff, and the evidence regarding reliability and efficacy seems poor. Exposed to venous obstruction, an SBT will within minutes become dark and discolored, and after pinprick, abundant, dark bleeding can be expected. Nevertheless, in a series of auto-SBT monitored clinically, 2 out of 37 transplants were lost due to venous ischemia [7]. The method can be enhanced by the use of telecommunications, as described by Chen et al. [9]. Yet, as repetitive evaluation of a stoma or a transfer placed intraoral increases the discomfort of the patient, it is tempting to reduce the frequency by which this monitoring procedure is performed.

### 3.2. Monitoring of an Exteriorized Segment

Completely buried SBTs can be visualized by an exteriorized segment based on the same vascular supply, a monitoring method first described by Katsaros et al. back in 1985 [10]. Schneider et al. published in 2006 their experiences using this monitoring method when performing 53 auto-SBTs [11]. They managed to salvage 1 SBT and in other 5 cases to perform replacement of a failing transfer with a new SBT. Two years later, in 2008, Bertino et al. reported how they used the same monitoring method to detect ischemia and harvest and insert a new auto-SBT in 3 of the 5 cases of POII [3]. It is interesting, though, that in 4 out of these 5 cases of POII the main symptom of flap failure was mouth bleeding [3]. 

### 3.3. Monitoring through a Window to the SBT

Several publications describe modified clinical monitoring methods by the use of an opening in the front of the neck region for inspection of the auto-SBT either directly, as described by Bootz and Müller [12], through a silastic sheeting, as described by Hester et al. [13], or through a split-skin transplant, as described by Bafitis et al. [14]. Skepticism regarding reliability and efficacy is even more pronounced using this method, as sensitivity is impaired due to fibrin and slough adhering to the exposed raw surface. 

### 3.4. Endoscopy

Monitoring for the detection of POII can be performed by endoscopy [15, 16]. This method is reliable for the evaluation of the transplant and, as biopsies can be harvested, outstanding for the detection of rejection as well. Yet, as the procedure itself is demanding with respect to equipment, specially trained personnel, and cooperation with the patient, it is not realistic to perform it more than 1 to 3 times a week. Nevertheless, the method might be excellent as a secondary challenge test to be performed if a less specific screening indicates POII.

### 3.5. Microendoscopy

Microendoscopy is on the one hand a direct visualization of the transplant and on the other hand a monitoring of the blood flow, as the method enables inspection of moving erythrocytes. Upile et al. argue that the method is of value intraoperatively as well as in the postoperative period, and that monitoring can be performed from the serosal or the mucosal surface of the transplant [17]. Yet, as with endoscopy, the procedure is very demanding and not suited to be performed with 1- or 2-hour intervals. It is definitively a method to be considered as a procedure for verification or disproval in case of suspicion of POII.

### 3.6. Echography

The peristalsis in the SBT can be visualized using echography as described by Yamada et al. in 2002 [18]. Yamada et al. argue that the method is simple, inexpensive, and noninvasive. In case of no peristalsis, the SBT can be stimulated through the overlying skin. Yamada et al. recommend monitoring by echography every 4 h during the first 3 postoperative days, but they recommend as well that other monitoring techniques and equipment are available in case peristalsis cannot be achieved [18]. 

## 4. Monitoring of the Blood Flow

This group of monitoring methods comprises all methods involving monitoring of the blood flow from the anastomosing vessels down to the capillaries and on the arterial and/or the venous side. 

### 4.1. Handheld Ultrasound Doppler

The handheld ultrasound Doppler is a noninvasive monitoring method [19]. Wright and Hobson II demonstrated back in 1975 the potential of the handheld ultrasound Doppler for the prediction of intestinal viability [20]. The method is reliable and fast when used during surgery directly on the tissue of interest. Later, the limitations of the method were described by Harrison et al. in 1981 [21], by Jones in 1992 [22], and by Stephnick and Hayden in 1994 [23]. The problem is the interference by other vessels in the adjacent tissue, an interference that can only be avoided if the exact location of the vessels to monitor can be stated. So, for postoperative surveillance to detect POII, the handheld ultrasound Doppler can be used in combination with an exposed part of the SBT, but is not recommended if central or peripheral vessels in the transplant cannot be identified. 

### 4.2. Laser Doppler

Laser Doppler monitoring is another noninvasive monitoring method, and the method allows for continuous monitoring [24]. Monitoring by laser Doppler is easy to perform and noninvasive, but the monitoring device has to be attached to the tissue of interest. Hallock and Koch solved this problem by combining the exteriorized segment of an auto-SBT with laser Doppler monitoring [25]. Obata et al. described in 1995 the supplement by a sensor-holding system that enabled the fixation of the laser Doppler flow meter to the surface of the exposed part of the intestine [26]. So, as with direct visualization, there is a need for an exposed part of the SBT, but the monitoring procedure is much more easily performed and causes the patient much less discomfort; it is also fast and can be carried out continuously.

### 4.3. Implantable Doppler

The implantable Doppler seems at a glance the most ideal solution for monitoring any auto- or allotransplant, as it is extremely fast and allows continuous monitoring [27]. In the study by Jones et al. 16 auto-SBTs were monitored by the use of the implantable Doppler, and the monitoring device was placed on the artery of the transplant [28]. They performed revision of 1 arterial anastomosis during surgery according to the Doppler sound, and in the postoperative monitoring period they reexplored 3 anastomoses, but all of them without ischemia. The problem of detecting the venous obstructions when placed on the artery was solved when Swartz et al. presented their next series, where the device was placed around the vein in 103 transplants [29]. They ended up saving 12 of the 16 primary failures detected. There might still, however, be a problem with specificity, especially in buried transplants. Rosenberg et al. reported in 2006 their experience using the implantable Doppler in 20 buried transplants (2 of which were auto-SBTs) [30]. They detected 8 alarms, but 7 of the 8 were false alarms. They saved the one ischemia-suffering flap, and there were no cases of neglected ischemia. Yet even if the implantable Doppler might be low in specificity, it still represents a fast and sensitive screening. Placement of the implantable Doppler at the vascular pedicle in an allo-SBT can be a challenge. The safest method will be to place the monitoring device around the artery, but in such a scenario venous ischemia will not be detected immediately. To obtain an early warning regarding venous congestion, the monitoring device has to be placed around the vein of the transplant, and with the thin wall of the visceral veins the placement itself might induce venous congestion. This problem might in an auto-SBT be solved by placing the device around the recipient vein in the neck region. This solution can only be used in cases where the venous anastomosis is performed end to end and not where it is performed end to side. 

### 4.4. Photoplethysmography

The blood flow in an SBT can be monitored by photoplethysmography, but an exposed part of the transplant is needed. Alos et al. demonstrated that photoplethysmography in a canine study showed a sensitivity and a specificity regarding survival of enteric anastomoses of 100% [31]. Katsaros et al. described back in 1985 how the nursing staff preferred the use of a photoplethysmography probe when observing the exteriorized segment of the auto-SBT, while the physicians preferred to note the color and to induce bleeding (simple clinical monitoring) [10]. As for the direct visualization as well as handheld and laser Doppler, this monitoring method requires an exposed part of the SBT.

## 5. Methods for Monitoring the Consequences of the Blood Flow

This group of methods covers all the techniques by which the consequences of a sufficient and/or inadequate blood supply to the SBT are monitored. 

### 5.1. Oxygen Content Monitoring

The content of oxygen in the SBT can be monitored either by monitoring the partial oxygen pressure (PO_2_) or by monitoring the percentage of tissue oxygen saturation of the hemoglobin (TOS). Driemel et al. described PO_2_ monitoring of auto-SBT in 2004 [32]. They demonstrated that the PO_2_ in SBTs is significantly higher than in musculocutaneous transplants. A modification of the photoplethysmographic monitoring (pulse oximetry), implying the possibility of monitoring TOS, has been described by Crerar-Gilbert et al. [33]. They obtained pulsative traces from nonoperated visceral tissue corresponding to the pulsative traces from the patient's finger, and they concluded that the method seems promising in terms of measuring the oxygenation of abdominal organs intra- and postoperatively. In 2005 Hirano et al. published a study investigating the TOS assessed by near-infrared spectroscopy in SBT in 12 pigs [34]. They found consistent values and could demonstrate significant differences between the central and the peripheral parts of the SBT. The methods seem sensitive and promising even though they require an exposed part of the SBT.

### 5.2. Carbon Dioxide Monitoring

Instead of monitoring the available oxygen, the accumulation of carbon dioxide can be monitored, as demonstrated by Imanishi et al. [35]. They monitored auto-SBTs in 20 patients. The monitoring was performed via an intraluminal balloon permeable to oxygen and carbon dioxide, and every half hour the content of the balloon can be withdrawn and analyzed. The disadvantages of the method include the delay in the answer and that the balloon obstructs the lumen of the SBT.

### 5.3. pH Monitoring

In an experiment Sheen et al. measured the pH of the mucosal surface of SBTs in 5 dogs [36]. They monitored pH directly, not indirectly via tonometry. They consistently found rapid changes within the first 10 minutes of arterial as well as venous ischemia. Corresponding significant and rapid changes in pH were published in 1994 by Yano et al. after an experimental study in rats [37]. Again they monitored directly, and they placed the micro-pH meter in the mesenterium of the SBT, thereby making it easier to keep in place. Hernandez et al. recommended pH monitoring as a very valuable tool to detect early POII after SBT when they published their canine experiment in 1996 [38]. Even though they used indirect monitoring of pH by tonometry and calculation by the use of the modified Henderson-Hasselbalch equation, they found fast and significant changes after arterial and venous ischemia. Kamiya et al. presented in 2007 clinical results after the indirect pH assessment of 35 auto-SBTs [39]. They found a good correlation between intramucosal pH and ischemic complications. Even though the method is noninvasive, it is not without discomfort for the patient, and monitoring was only performed at 6- to 12-hour intervals. pH monitoring seems promising, as it shows significant and objective changes in case of ischemia. Despite minimal invasiveness, the direct method of monitoring seems favorable to the indirect tonometry assessment, as it can be performed continuously and without noteworthy discomfort for the patient. Further evolution of the micro-pH meter needs to be performed before the method can be introduced clinically in SBT monitoring.

### 5.4. Monitoring of Metabolism by Microdialysis

In 1999 Tenhunen et al. reported that microdialysis can be used for the detection of intestinal ischemia by monitoring metabolic markers [40]. High sensitivity and specificity have been proven experimentally [41], and likewise clinical reliability has been demonstrated in monitoring auto-SBT [42]. The method seems promising, as it is minimally invasive, objective, and does not cause discomfort for the patient or require an exposed part of the transplant [43]. The weakness of the method is the time lag, but Deeba et al. have shown that this problem can be solved [44]. 

## 6. Discussion

The ideal monitoring method for the detection of POII ischemia after SBT should be

1. reliable and fast,
2. minimally invasive or noninvasive,
3. safe,
4. objective,
5. easy to apply, read, and remove,
6. cheap,
7. without significant discomfort for the patient.

The choice of whether to perform surveillance regarding POII depends very much on the risk of POII and the consequences in the case of neglected POII. The risks as well as the consequences are not the same in auto- and allo-SBT, and they will vary from center to center and from one group of patients to another. The possibilities of action in case POII is detected also have to be considered. When an auto-SBT is found ischemic, an immediate retransplant with a second SBT is a possibility if the first cannot be salvaged. When an allo-SBT is found ischemic, the only chance is to either salvage the transplant or to remove it to salvage the patient. 

When considering which monitoring method to use, the risk of false negative and false positive alarms has to be discussed. In clinical monitoring the sensitivity and specificity of a monitoring method originating from an experimental study definitely is important. Nevertheless, clinical monitoring is not as standardized as experimental monitoring, and in the daily routine there will be situations, which have never even been thought of in experimental settings. Similarly, unexpected discomfort for the patient can be a matter of huge importance in the clinical assessment of a monitoring method, whereas this has not been an issue at all while the method has been tested in several experimental trials. 

Without a doubt, the optimal way to find out which monitoring method to use would be a large randomized controlled trial (RCT) with +/− monitoring and survival of the transplant and the patient as the primary endpoints. Unfortunately such a study will not be performed. Due to the small numbers of POII, the RCT will require more than 1000 participants with SBT, and as several of the monitoring methods described here are under evolution, these methods will have changed before the trial can be completed. 

There is no hardcore evidence supporting performance of surveillance to detect POII after SBT. Therefore, it can be considered inappropriate to do so. On the other hand, neither is there evidence saying that surveillance should not be performed. And it definitively can be judged inappropriate not to monitor the transplant and the patient. Despite the lack of guidelines regarding procedures when SBT is performed, patients all over the world are operated on with auto- and allo-SBT today. Therefore, all positive and negative experiences are to be considered when decisions are taken. Consequently, it is important that all knowledge is published and thereby becomes available as a foundation for these decisions. Another aspect to consider when deciding which procedure to use is the need for organ donation and living donors. It can be expected that knowledge about maximum care and monitoring of every single intestinal transplant will have a positive impact on potential donors.

## 7. Conclusion

Several monitoring methods are available for the detection of postoperative intestinal ischemia in small bowel transplants. No ideal monitoring method has been found, and no documentation obtained has proven the benefit of surveillance, and for that reason no general guidelines can be formulated. Whether to implement monitoring and which method to choose are choices taken at each center performing auto- and/or allo-transplant of the small bowel. 

## References

[1] Daiko H, Hayashi R, Saikawa M (2007). Surgical management of carcinoma of the cervical esophagus. *Journal of Surgical Oncology*.

[2] Ruiz P, Kato T, Tzakis A (2007). Current status of transplantation of the small intestine. *Transplantation*.

[3] Bertino G, Benazzo M, Occhini A, Gatti P, Spasiano R, Alessiani M (2008). Reconstruction of the hypopharynx after free jejunum flap failure: is a second free jejunum transfer feasible?. *Oral Oncology*.

[4] Lauro A, Di Benedetto F, Masetti M (2005). Twenty-seven consecutive intestinal and multivisceral transplants in adult patients: a 4-year clinical experience. *Transplantation Proceedings*.

[5] Siniscalchi A, Piraccini E, Miklosova Z (2007). Metabolic, coagulative, and hemodynamic changes during intestinal transplant: good predictors of postoperative damage?. *Transplantation*.

[6] Zanfi C, Cescon M, Lauro A (2008). Incidence and management of abdominal closure-related complications in adult intestinal transplantation. *Transplantation*.

[7] Robinson DW, MacLeod A (1982). Microvascular free jejunum transfer. *British Journal of Plastic Surgery*.

[8] Lahteenmaki T, Pukander J, Isolauri J, Waris T (1992). Pharyngo-oesophageal reconstruction with free jejunal transplants. New design of the upper anastomosis to improve monitoring of viability of transfer. *Scandinavian Journal of Plastic and Reconstructive Surgery and Hand Surgery*.

[9] Chen H, Kuo H, Chung K, Chen S, Tang Y, Su S (2012). Quality improvement of microsurgery through telecommunication? the postoperative care after microvascular transfer of intestine. *Microsurgery*.

[10] Katsaros J, Banis JC, Acland RD, Tan E (1985). Monitoring free vascularised jejunum grafts. *British Journal of Plastic Surgery*.

[11] Schneider NK, Koehler C, Wedler V, Kuenzi W (2006). Oropharyngeal reconstruction with a free jejunum graft after tumour and stenosis resection: an analysis of 53 cases. *Handchirurgie Mikrochirurgie Plastische Chirurgie*.

[12] Bootz F, Müller GH (1988). Postoperative monitoring of a free jejunum transplant. *Laryngologie, Rhinologie, Otologie*.

[13] Hester TR, McConnel FMS, Nahai F (1980). Reconstruction of cervical esophagus, hypopharynx and oral cavity using free jejunal transfer. *American Journal of Surgery*.

[14] Bafitis H, Stallings JO, Ban J (1989). A reliable method for monitoring the microvascular patency of free jejunal transfers in reconstructing the pharynx and cervical esophagus. *Plastic and Reconstructive Surgery*.

[15] Pavlovics G, Cseke L, Papp A, Tizedes G, Tabar BA, Horvath PO (2006). Esophagus reconstruction with free jejunal transfer. *Microsurgery*.

[16] Sandrasegaran K, Lall C, Ramaswamy R (2011). Intestinal and multivisceral transplantation. *Abdominal Imaging*.

[17] Upile T, Jerjes W, El Maaytah M, Hopper C, Searle A, Wright A (2006). Direct microvascular monitoring of a free autologous jejunal flap using microendoscopy: a case report. *BMC Ear, Nose and Throat Disorders*.

[18] Yamada N, Kakibuchi M, Kitayoshi H (2002). An easy method for monitoring free jejunal transfer by detecting peristalsis using echography. *European Journal of Plastic Surgery*.

[19] Franklin DL, Schlegel W, Rushmer RF (1961). Blood flow measured by Doppler frequency shift of back-scattered ultrasound. *Science*.

[20] Wright CB, Hobson RW (1975). Prediction of intestinal viability using doppler ultrasound technics. *American Journal of Surgery*.

[21] Harrison DH, Girling M, Mott G, Acland RD (1981). Experience in monitoring the circulation in free-flap transfers. *Plastic and Reconstructive Surgery*.

[22] Jones NF (1992). Intraoperative and postoperative monitoring of microsurgical free tissue transfers. *Clinics in Plastic Surgery*.

[23] Stepnick DW, Hayden RE (1994). Postoperative monitoring and salvage of microvascular free flaps. *Otolaryngologic Clinics of North America*.

[24] Svensson H, Pettersson H, Svedman P (1985). Laser Doppler flowmetry and laser photometry for monitoring free flaps. *Scandinavian Journal of Plastic and Reconstructive Surgery*.

[25] Hallock GG, Koch TJ (1990). External monitoring of vascularized jejunum transfers using laser Doppler flowmetry. *Annals of Plastic Surgery*.

[26] Obata T, Hirata T, Yamanaka Y, Uchida Y (1995). Blood flow rate in jejunal ischemia-reperfusion injury. *Experientia*.

[27] Swartz WM, Jones NF, Cherup L, Klein A (1988). Direct monitoring of microvascular anastomoses with the 20-MHz ultrasonic Doppler probe: an experimental and clinical study. *Plastic and Reconstructive Surgery*.

[28] Jones NF, Rocke AM, Swartz WM, Klein A (1989). Experimental and clinical monitoring of free jejunal transfers using an implantable ultrasonic Doppler probe. *British Journal of Plastic Surgery*.

[29] Swartz WM, Izquierdo R, Miller MJ (1994). Implantable venous doppler microvascular monitoring: laboratory investigation and clinical results. *Plastic and Reconstructive Surgery*.

[30] Rosenberg JJ, Fornage BD, Chevray PM (2006). Monitoring buried free flaps: limitations of the implantable Doppler and use of color duplex sonography as a confirmatory test. *Plastic and Reconstructive Surgery*.

[31] Alos R, Garcia-Granero E, Calvete J, Uribe N (1993). The use of photoplethysmography and Doppler ultrasound to predict anastomotic viability after segmental intestinal ischaemia in dogs. *The European Journal of Surgery*.

[32] Driemel O, Oberfahrenhorst I, Hakim SG, Kosmehl H, Pistner H (2004). Intra- and postoperative monitoring of transplanted flaps. Measurement of the partial pressure of oxygen in tissue. *Mund Kiefer Gesichtschir*.

[33] Crerar-Gilbert AJ, Kyriacou PA, Jones DP, Langford RM (2002). Assessment of photoplethysmographic signals for the determination of splanchnic oxygen saturation in humans. *Anaesthesia*.

[34] Hirano Y, Omura K, Yoshiba H (2005). Near-infrared spectroscopy for assessment of tissue oxygen saturation of transplanted jejunal autografts in cervical esophageal reconstruction. *Surgery Today*.

[35] Imanishi Y, Nameki H, Isobe K (2003). Intramucosal PCO2 measurement as a new monitoring method of free jejunal transfer following pharyngo-laryngo-esophagectomy. *Plastic and Reconstructive Surgery*.

[36] Sheen R, MacLeod AM, O’Brien BM (1987). Intraluminal pH measurement to monitor the vascular perfusion of free jejunal autografts: an experimental study. *Australian and New Zealand Journal of Surgery*.

[37] Yano K, Hata Y, Matsuka K, Ito O, Matsuda H (1994). Intestinal tissue pH monitoring in microsurgery: an experimental study in rats. *Annals of Plastic Surgery*.

[38] Hernandez G, Lopez F, Castillo L (1996). Postoperative ischemia after gut transplantation: role of different monitoring systems. *Transplantation Proceedings*.

[39] Kamiya K, Suzuki S, Mineta H, Konno H (2007). Tonometer pHi monitoring of free jejunal grafts following pharyngolaryngoesophagectomy for hypopharyngeal or cervical esophageal cancer. *Digestive Surgery*.

[40] Tenhunen JJ, Kosunen H, Alhava E, Tuomisto L, Takala JA (1999). Intestinal luminal microdialysis: a new approach to assess gut mucosal ischemia. *Anesthesiology*.

[41] Birke-Sorensen H, Andersen NT (2010). Metabolic markers obtained by microdialysis can detect secondary intestinal ischemia: an experimental study of ischemia in porcine intestinal segments. *World Journal of Surgery*.

[42] Sorensen HB (2008). Free jejunal flaps can be monitored by use of microdialysis. *Journal of Reconstructive Microsurgery*.

[43] Sommer T (2005). Microdialysis of the bowel: the possibility of monitoring intestinal ischemia. *Expert Review of Medical Devices*.

[44] Deeba S, Corcoles EP, Hanna BG (2008). Use of rapid sampling microdialysis for intraoperative monitoring of bowel ischemia. *Diseases of the Colon and Rectum*.

